# College students’ attachment to their smartphones: a subjective operant approach

**DOI:** 10.1186/s40359-022-00857-x

**Published:** 2022-06-08

**Authors:** Zixue Tai, Cheng Dai

**Affiliations:** 1 School of Journalism and Media, University of Kentucky, Lexington, USA; 2 School of Journalism and Communication, Minjiang University, Fuzhou, China

**Keywords:** Mobile attachment, Q methodology, College students, Attachment theory, Smartphone use

## Abstract

**Background:**

Smartphone use has become a pervasive aspect of youth daily life today. Immersive engagement with apps and features on the smartphone may lead to intimate and affectionate human-device relationships. The purpose of this research is to holistically dissect the ranked order of the various dimensions of college students’ attachment to the smartphones through the by-person factorial analytical power of Q methodology.

**Methods:**

Inspired by extant research into diverse aspects of human attachment to the smartphones, a concourse of 50 statements pertinent to the functional, behavioral, emotional and psychological dimensions of human-smartphone attachment were pilot tested and developed. A P sample of 67 participants completed the Q sort based on respective subjective perceptions and self-references. Data was processed utilizing the open-source Web-based Ken-Q Analysis software in detecting the main factorial structure.

**Results:**

Five distinct factor (persona) exemplars were identified illustrating different pragmatic, cognitive and attitudinal approaches to smartphone engagement. They were labeled mainstream users, disciplined conventionalists, casual fun-seekers, inquisitive nerds, and sentient pragmatists in response to their respective psycho-behavioral traits. There were clear patterns of similarity and divergence among the five personas.

**Conclusion:**

The typological diversity points to the multiplicate nature of human-smartphone attachment. Clusters of cognitive, behavioral and habitual patterns in smartphone engagement driving each persona may be a productive area of exploration in future research in exploring their respective emotional and other outcomes. The concurrent agency of nomophobia and anthropomorphic attribution is an intriguing line of academic inquiry.

## Background

The centrality of mobile media in the fabric of daily routines has become a hallmark of contemporary youth culture. The broadening scope of immersive engagement with the smartphone has cultivated heterogeneous mobile lifestyles among the current youth generation [1, 2]. Granted, the use of the ever-expanding list of smartphone features fulfills Maslow’s hierarchical needs among users across different cultures [3, 4], and the smartphone has evolved into an anchor device for millennials to multitask and consume multiplatform media [5]. Moreover, perpetual contact with the mobile phone expectedly results in varied forms of intimate and affectionate relationships between the user and device. There is therefore the need for researchers to move beyond the functional, applicational, and behavioral aspects of use patterns and user traits in dissecting the emotional and psychological dimensions of smartphone engagement and their subsequent outcomes.

One conceptual model in understanding the psychological and emotional aspects of smartphone engagement is mobile attachment. Attachment theory, which originated in the work by John Bowlby [6], argues that human beings need to build a close and continuous supportive relationship with their caregivers in order to thrive socially and emotionally. It distinguishes two forms of caregiving processes in interpersonal relationships: safe haven and secure base. The former responds timely and flexibly to the care receiver’s imminent need for comfort and assistance, whereas the latter provides available support when called upon for the attached person’s personal growth and exploration while venturing out into the external world [7]. Attachment was taken forward by Ainsworth and colleagues, who, through their “Strange Situation Procedure,” identified another form of attachment, namely attachment insecurity (often manifested in symptoms of anxiety and avoidance) [8]. Insecurity has been found to be significant predictors of addictive behavioral tendencies toward the smartphones [9] and social networking sites [10]. Increasing dependence on the smartphones has derived not only functional benefits but also affective outcomes such as stress relief and emotional comfort, leading the smartphone to be perceived as a “pacifying technology” by users [11, 12]. Indeed, use of the smartphone as a an attachment target has been found to be an important predictor for problematic smartphone use [13].

The embedding of smartphone-based personalized communication in multifarious sentient environments creates facets of experience in extending one’s digital self [14–16]. The smartphone extends one’s sense of self through channeling and modulating a symbiotic relationship with identity, social and romantic relationships, agency, and experienced authenticity in the physical world [17]. Due to its immense popularity and ubiquity, smartphone has attracted mounting attention from researchers as object of human attachment in recent years. Toward this end, Wehmeyer pinpointed three dimensions of user-device attachment to the mobile phones: *symbolism* (e.g. social status, group membership or other personal meanings), *aesthetics* (e.g. design features and visual and personalized appearances), and *necessity* (e.g. fulfilling everyday acts of communicating and socializing) [18]. Likewise, Park and Kaye’s research identifies three types of extension of the self via the smartphone: functional extension (instrumental capabilities), anthropomorphic extension (humanlike attributes and traits), and ontological extension (emotional outcomes) [19–21].

The anthropomorphism– typically manifested in personalization and personification—of the smartphone has also attracted significant scrutiny from researchers. For its capabilities to enable affectionate bonding with users, the smartphone has been conceived as “digital companions” [22, 23] and “virtual friends” [24]. A sample of British adults are characterized as *homo prostheticus* in developing an intercorporeal relationship in the form of human-smartphone assemblage intermingling the body and the machine [25]. Three social dispositional factors have been identified as contributing to the awareness and acceptance of anthropomorphisization of the smartphones by college students: social disposition, attachment style, and cultural orientation [26].

Seamlessly consolidating computing, portability, and mobility into one interface, smartphone is prone to problem use, which may lead to various negative outcomes. Research in cross-national settings has identified a multiplicity of technological paradoxes that demonstrate conflict and tension along opposing dimensions such as empowerment/enslavement, independence/dependence, competence/incompetence, engaging/disengaging, public/private, and illusion/disillusion [27–29]. While smartphones may facilitate everyday life, they can also create “technoference”—intrusions upon and disruptions to interpersonal relationships and individual well-being [30, 31]. Excessive dependency on the smartphone in everyday routines has led to a new type of disorder called *nomophobia* (no mobile phone phobia), defined as the fear or anxiety over the temporary inability to use or reach one’s mobile phone [32–35]. Another tendency that breaches conventional social norm is *phubbing*—describing the act of an individual checking the smartphone and hence snubbing others while in a social setting—a behavior that has been found to be especially prevalent among adolescents and young adults [9, 36–38]. Moreover, smartphone use, through its design features and content type, is habit-forming [39, 40]. Certain habitual patterns are leading causes of addictive and pathological behaviors [41, 42], and research examining smartphone addiction in its various forms and causes has been a productive area of scholarly effort in recent years [43–50].

It is worth highlighting that research endeavors and theoretical perspectives as reviewed above have been dominated by the variable-centric interindividual differential approach of R (i.e. attribute-based by-variable correlational and/or factorial techniques). Besides, the scope of inquiry has been heavily oriented toward a compartmentalized focus specializing in one or a small number of chosen areas of researchers’ interest. One notable exception is the Smartphone Impact Scale (SIS) by Pancani et al. in assessing behavioral, social, affective and cognitive impact through human-smartphone interaction along seven dimensions [51]; there are also efforts to measure smartphone attachment utilizing multi-dimensional items in cross-sectional survey settings [52, 53]. Our study offers competing and complementary insight on user-smartphone relationship by utilizing the Q methodology which, first developed by Stephenson (1953) as a “inverted” by-person factor analytical approach, captures *intra-individual* variations and *holistic* personified viewpoints as rendered through its sophisticated statistical rigor of *structured, forced-choice ranking* of subjective statements.

## Methods

### Q methodology

Q methodology was nurtured by Stephenson as “a science of subjectivity” where individual perspectives, discourses and opinions become the locus of inquiry [54], It provides a robust, natural epistemic framework to disentangle “discursive complexity” [55], and to “discern people’s perceptions of their world from the vantage point of self-reference” [56]. As a hybrid, or a mixed methods approach [57], Q combines qualitative focuses with quantitative statistical procedures in pattern-making among respondents based on individual profiles and subjective interpretations [54, 58, 59]. This “qualiquantology” provides Q the “perturbating power” in dissecting multiplex issues involving human subjectivity [60]. Among the defining features of Q are “its focus on the subjective experiences of participants, its emphasis on context, and its privileging of the everyday and local” as well as “its capacity to subvert the power of the researcher” [55]. The last point is echoed by Goldman as a “paradigmatic synthesis of the human and natural sciences models of inquiry” due to Q’s incorporation of “mathematical-statistical empiricism” in its analysis [61]. As a result, Q is advocated as “a much more objective way to garner knowledge other than qualitative methods” [55] throuch which “subjective input produces objective structures” [62].

Q methodology was developed at the moment when correlational and factor analytical approaches—spearheaded by Karl Pearson and Charles Spearman—had established themselves as the dominant paradigm of traditional psychology and behavioral sciences [63]. Because Pearson’s r provides the foundation for these statistical procedures, Stephenson called these approaches R methodology, in contrast to his Q methodology [54]. Whereas R takes the objectivist stance of detecting individual variations through measurement of psychometric attributes and personal traits using samples of people, Q adopts the subjectivist approach of pinpointing differing perspectives and attitudes through analyzing the concourse of communication as implemented in samples of opinionated statements [58]. In other words, the focus of inquiry for Q is the “constructions,” vis-à-vis that of “constructors” in R [63].

The utility of Q has been amply demonstrated in diverse contexts across disciplines. An edited volume by Addams and Proops (2000), for example, shows the breakthrough Q methodology can bring to the “interwoven complexity of environmental beliefs and attitudes” by the affected publics, which leads them to conclude that “Q may be almost a perfect technique in the initial stages of environmental policy analysis” [64]. Q analysis of subjective perspectives leads to a typology of social media users as regards information control and presentation in one study [65], and developed a theoretical typology of digital news consumption/reception in another [66]. Following similar methodological trajectories, our study aims to explore the “gestalt configurations” [62] of the meanings and value college students assign to their smartphones.

### Research questions, Q sample and participants (P set)

One commonly acknowledged advantage of Q methodology is that it offers a “more macroscopic” complement to conventional qualitative approaches [62], and excels in covering a wide range of viewpoints favored or shared by a specific group of participants [55, 57]. On the other hand, its inherent antithesis of hypothetico-deductivism [54] makes it appropriate in bringing coherent answers to broadly stated research questions rather than test hypotheses, which is congruent to Brown’s recommendation to Q researchers in attributing meaning “a posteriori through interpretation rather than through a priori postulation” [58]. In broad strokes, our research questions are aligned along diverse aspects of functional, applicational, habitual, behavioral, anthropomorphic, emotional dimensions of smartphone use. One distinct vantage point proffered by Q allows us to discern the relative weight informants assign to the various dimensions of human-device attachment not available in typical research studies dedicated to a narrower (variable-centric) scope of interrogation.

A core tool in Q methodology is the *concourse of communication*, which refers to “the volume of discussions about a topic” and supplies the “raw material” (Q sample) [56]. In conformance with the established practice in Q methodology research [56, 59, 63], we developed the initial concourse of statements for our research after an extensive review of academic publications pertaining to diverse aspects of mobile attachment. Special attention was paid to subjective items and attitudinal tendencies that partake of parameters of user-device attachment in the broad context of functional features, behavioral patterns, psychological traits, and emotional attributes as mentioned in the literature reviewed in the Background section. We then explored the applicability and appropriacy of these items by pilot testing them through interviews and surveys with 23 students by asking them to judge the relevancy of each item to their self-reference on a 1–5 scale. Among the 75 initial statements, these that were not ranked at all, or ranked very low in our pilot test were dropped from the list, and a few items that students indicated as important to them were added. We narrowed the Q set sample to a total of 50 statements, to be sorted in the scheme detailed in Table 1.

**Table 1:** Forced-choice Q distribution

Ranking value	− 5	− 4	− 3	− 2	− 1	0	+ 1	+ 2	+ 3	+ 4	+ 5
Number of items	2	3	4	6	6	8	6	6	4	3	2

### Research procedure and Q analysis

After ethics approval was granted, we recruited participants (P set) through written informed consent from a public university in southeastern China. Participation in the study was voluntary, and students were allowed to discontinue at any moment, or to skip any statements they did not feel comfortable with ranking. Conforming to standard Q procedure, each statement was printed on an index card, and participants were instructed to lay out all statements on a table. They were first asked to group the statements into three broad categories: those that they disagreed with; those that they agreed with; and those that they could not decide. They were then asked to start matching statements to the corresponding numbered slots on the Q Sort Table from the statement they most disagreed with (− 5), followed by the statement they most agreed with (+ 5), and so on, until they had completed sorting all statements. They were allowed to change their ranking in the process, until they felt comfortable about all the choices at the end. Each participant completed this process at their own pace, without discussion with anybody.

After all Q statements were sorted, we asked participants to provide open-ended responses in expounding on rationales driving their sorting decisions, and offer additional perspectives on their personal engagement with the smartphones. This additional process generated comments that help aid, clarify, or verify interpretations [58, 62], thus enhancing “accuracy and efficacy” of the respective factor arrays [55]. All but one student provided (varyingly) detailed information behind their decision-making. We follow the recommended procedure of focusing on input from participants who display a significant factor loading in the ensuing analysis as an effort to detect consistent thematic lines and discursive patterns within each persona [56, 62, 67]. Responses to open-ended questions were analyzed by following the epistemological framework and procedural guidelines of thematic analysis in identifying and organizing content meaning into salient threads and patterns [68].

In the last stage of the study, each student was asked to provide demographic information (e.g., gender, age, major), and then to browse their phone for a few minutes by browsing their Screen Time and app use statistics, with these questions in mind: What apps did they use in the prior week, and how often? They then provided responses to items in a questionnaire inquiring into these issues. A total of 75 students took part in the study, in five separate groups; the process took each participant 50 min to slightly over one hour. We conducted t-test in comparing genderwise differences regarding app use. Due to the relatively small size of the participant pool (20 males vs 47 females), we adopted a threshold value of *p* < 0.05 in reporting our results.

Eight of the completed Q sorting tables were tossed out due to missing or duplicate items, and our subsequent analysis is based on 67 valid responses (47 female versus 20 male). We used the open-source Web-based Ken-Q Analysis software version 1.06 (https://shawnbanasick.github.io/ken-q-analysis/) in performing the various statistical procedures, with the main by-person factor analysis performed through principal component analysis (PCA) using Varimax rotation. The focus was placed on four areas as commonly highlighted in Q analysis [56–58, 67]: factor scores, representative sorts for each factor, distinguishing statements, and consensus statements.

## Results and findings

First of all, as revealed in Table 2, students’ use of smartphone-based apps is encompassing and wide-ranging, covering functions from instant messaging, chatting, mobile payment, e-shopping, entertainment, information-seeking and navigation. Worth highlighting is the diminished role of conventional voice call and text messaging, ranked #15 and #19 respectively out of 23 apps. Not long ago, these had been consistently found to be two of the most profusely used features in mobile services [27, 69, 70]. These are clear signs that the smartphone has profoundly changed the landscape of mobile communication. The students reported an average of close to seven hours (male = 6.6 vs. female = 6.7) on weekdays and over nine hours (male = 9.0 vs. female = 9.2) on weekend days. Genderwise t-test analysis shows that, in comparison with their male peers, female students tend to display the following patterns: have fewer regular contacts; use more Weibo and WeChat; shop substantially more on Taobao; rely more on the photo-editing app Ulike; make more e-payments via Alipay (probably due to making more online purchases).

**Table 2:** Frequency of app use (N = 69) (1 = Rarely; 2 = Occasionally; 3 = Sometimes; 4 = Often; 5 = Daily)

App (utility)	Mean	SD
QQ (instant messaging)	4.88	0.54
Alipay (mobile payment)	4.64	0.73
WeChat (social networking)	4.49	0.94
Clock (time-keeping)	4.37	1.13
Sina Weibo (microblogging)	4.03	1.15
Baidu (search engine)	3.94	1.01
NetEase cloud music (music streaming)	3.81	1.37
ELEME (online food delivery)	3.78	1.31
Taobao (online shopping)	3.73	1.02
Weather (weather forecast)	3.67	1.28
(Built-in) camera (photo-taking)	3.66	1.05
bilibili (video sharing)	3.33	1.40
Reminders (planner & calendar)	2.88	1.15
Meituan (location-based E-commerce)	2.76	1.22
Voice call (conventional phone call)	2.75	1.12
Aiqiyi (online video)	2.55	1.33
TikTok (video-focused social networking)	2.31	1.42
Didi (ride-hailing)	2.25	0.91
SMS (mobile phone-based text messaging)	2.10	1.08
Ulike (photo retouching)	2.09	1.15
NetEase youdao (online dictionary & information seeking)	2.09	1.26
RED/Xiaohongshu (social media & E-commerce hybrid)	1.96	1.21
Toutiao (news & information updates)	1.93	1.11

By-person factor analysis yielded a five-factor typological structure. Composite reliability, which measures the correlation of flagged Q sorts among participants in the same factor, ranges from 0.988 (Factor 1) to 0.923 (Factor 4), thus pointing to a high level of congruence among individuals within each factor. We associate each factor with a particular persona, by incorporating factor scores/loadings, and the “best approximation” [60] or the “most representative sorts” [67] corresponding to each factor. Table 3 reports the details of the item rankings for each of the five “factor exemplars.”

**Table 3:** Factor arrays for the five factors

Statement	F1	F2	F3	F4	F5
S29. My smartphone holds lots of my personal secrets. I would feel very uncomfortable if somebody looked inside my phone	+ 5	+ 1	0	+ 5	+ 1
S20. I am used to smartphone browsing prior to going to bed. If I don’t do this, I have a hard time getting to sleep	+ 5**	− 1	+ 1	− 1	0
S40. I feel safe when I have my smartphone with me. It will help me at times of emergency	+ 4	+ 2	+ 2	+ 3	+ 3
S14. I habitually check my smartphone once every few minutes. If I don’t check the phone for an extended period of time, I feel like I am missing something important	+ 4**	0	0	0	− 2
S10. My smartphone is my wallet, my notebook, and my backpack—I need it to make payment, take notes, and record important information	+ 4	+ 4	+ 4	+ 5	+ 5
S45. When I get onto my smartphone, I often don’t realize how quickly time goes by	+ 3	+ 2	+ 3	+ 1	+ 1
S2. My smartphone is my main tool of entertainment. I use it to watch videos, read books, and enjoy music	+ 3	+ 5	+ 5	+ 2	+ 5
9. Whenever I have free time, I get onto my smartphone to kill the time	+ 3	+ 2	+ 4*	+ 1	+ 3
47. After having studied hard or having worked hard at something, it is a great joy to get onto my smartphone for casual browsing	+ 3	+ 4	+ 3	+ 2	+ 2
17. I feel embarrassed when people sit together and everyone is busy checking their smartphones, without anyone talking to each other	+ 2	+ 3	+ 1*	− 1*	+ 3
24. If I leave my phone behind, I will feel uneasy, and must go back to get my phone before I can continue with the rest of the day	+ 2	0	− 3	− 2	+ 3
33. When I buy a new smartphone, I mainly consider best value for the money and practical features. I won’t buy a pricey phone if there is a cheaper replacement available	+ 2	+ 4	+ 2	+ 4	+ 1
12. I often regret the excessive amount of time I spend on my smartphone, which is interfering with my routine study and daily life	+ 2**	+ 5**	0	0	0
48. I am used to casual browsing on my smartphone, just dawdling away my time for no particular purpose	+ 2	+ 1	+ 5**	− 2**	+ 1*
11. I have this hate-and-love feeling about my smartphone, because I cannot resist its temptation, and sometimes do things that I regret	+ 2*	+ 3*	− 2	0	0
50. My smartphone is my reliable helper when I have questions in my life and study	+ 1	+ 2	+ 4	+ 1	+ 4
23. I put my smartphone within easy reach. If it falls out of my sight, I will feel uneasy	+ 1	− 2**	-5**	+ 1	+ 2
1. My smartphone is like my housekeeper and aide. It helps me coordinate and track everyday things	+ 1	+ 1	+ 3	+ 3	+ 4
25. I keep my power bank with me all time. Otherwise my phone would run out of battery before day’s end	+ 1	− 3*	− 1	0	− 1
26. If I lost my smartphone, it would be disastrous. I would try everything possible to get it back before I could do anything else	+ 1	− 2	− 2	0	+ 2
16. When having a face-to-face conversation, I am offended if the other party checks the phone while listening to me talk	+ 1	+ 3	+ 1	0	+ 4
46. My smartphone gives me a platform to present myself. I use it to record important occasions in my daily life, and publicize information among my network of friends	0*	+ 1	+ 2	+ 4	+ 2
32. It is important for me to personalize my apps and the smartphone screen, because I consider my smartphone part of myself	0	+ 1	− 3**	+ 2	+ 1
43. I often get to the end of my bandwidth allowance, and sometimes I use up all the bandwidth at the end of the month	0	− 2*	+ 2*	0	− 4*
22. I am used to checking my smartphone while waking up in the middle of the night	0**	− 3	− 3	− 3	− 3
8. My smartphone is like my personal agent. It takes care of my daily routines; without it, I would not know what to do	0	− 4*	+ 1	− 1	+ 2**
27. If the teacher asks me to turn in my smartphone in class, I cannot help but think of my phone from time to time in class	0	− 1	− 1	− 1	− 1
44. I look upon my smartphone as my pal, who understands me well and keeps me company all the time	0	− 1*	+ 2**	− 5**	0
28. The smartphone is merely a tool for me. Even if I don’t have it, I believe I can still complete my daily study and living tasks	0	+ 2	+ 3*	+ 1	0
30. I am versed in knowing the features and using most of the apps on my smartphone	− 1	0**	− 1	+ 3**	− 2
15. With the smartphone on my side, I often have difficulty concentrating on the lectures or doing what the teacher asks me to do in class	− 1*	0**	− 4	− 2	− 2
6. My smartphone is like my right-hand pal. I cannot accomplish anything without its help	− 1	− 5	− 3	− 4	0
49. After having spent so much time with my smartphone, I sometimes feel I am further distanced from my friends and the outside world	− 1*	+ 1**	− 4	− 4	− 2
42. I often make an effort in reducing the amount of time I spend on my smartphone so as to reduce my dependency on it and minimize its interference with my daily life	− 1	+ 2	− 1	+ 2	− 1
41. Every time I buy a new phone, I feel emotional to part ways with my old phone	− 1	0	+ 2	0	0
7. I consider my smartphone a part of my extended family that promotes mutual understanding among family members	− 2	− 3**	+ 1	− 1	+ 2*
21. I feel that the use of smartphone has reduced the daily communication between me and my friends	− 2	+ 3**	− 2	− 4	− 2
4. My smartphone is like a naughty sister/brother: it is lovely when it behaves, but it can be disappointing and annoying at times when it does not behave	− 2	− 1	+ 1	− 2	0
5. My smartphone is like my pet—it knows how to please me, and it knows how to not upset me	− 2	− 4	0*	− 2	− 1
18. Everything I do now depends on the smartphone. I feel hard to communicate with people without the smartphone	− 2	− 2	− 4	− 3	− 1
31. My smartphone is like my twin sister/brother. I share everything with him/her, and she/he knows everything about me	− 2	− 3	− 1	− 2	− 2
3. I regard my smartphone as my slave/servant. It listens to me and does what I ask it to do	− 3	− 5	0**	− 3	− 3
19. My smartphone is just a tool for me in communicating with others. Except voice calls and WeChat messages, I rarely use other apps and features	− 3	− 2	− 2	− 5	− 5
35. I often search for new apps and study their new features, and try them out to see how it works	− 3	0	0	+ 2**	-3
38. I know more about smartphone technologies and apps than most of my fellow students	− 3	0	0	+ 2**	− 3
34. I pay attention to the latest developments with apps, and want to be among the first in installing updates	− 4	− 1*	0*	+ 4**	− 4
37. I pay attention to the newest smartphone releases, and want to know the latest technical features and applications	− 4	− 1	− 2	+ 2**	− 4
13. I maintain a good level of self-control, and typically complete the tasks I have planned for the day by not spending an excessive amount of time on smartphone use	− 4**	0	− 1	− 1	− 1
36. I value the brand of the smartphone I use, and want to get the best phone I can afford	− 5	− 2	− 5	− 3	+ 1**
39. Whenever a new generation of the smartphone is released, I try to upgrade to the latest phone if possible	− 5*	− 4	− 2*	+ 1**	− 5

Underlined items are consensus items among all five factors

*Distinguishing items, significant at *p* < 0.05

**Distinguishing items, significant at *p* < 0.01

### Most consensual and disagreed statements

The Q analysis yields a ranked order of the statements, from the most agreed to the most disagreed based on the sorting values of all participants. We selected 11 items in each category based on the Z-scores and patterns of inter-factor variation. The most consensual statements, whose Z-score values range from 0.011 (S27) to 0.164 (S9), reflect the degree of congruence among the five personas. They uniformly agree that if the teachers ask them to turn in their phones during class, they will not think of the phone during class (Statement 27, or S27); and having the phone with them brings a sense of safety (S40). They are disinclined to consider the phone as an intimate companion (31), and they consider their smartphone more than a tool of communication (S19). The students relish the joy of browsing the phone as a way to relax after some drudgery or menial tasks (S47). They don’t quite see the phone as negatively affecting their ability to communicate with others (S18), and they use the smartphone as a main tool of daily entertainment (S2). They all think highly of the smartphone as a wallet/notebook (S10), and as a personal assistant (S1). They do not check the phone in the middle of the night (S22), nor do they feel emotional in bidding farewell to their old phone (S41). A habitual practice by most is to switch the phone on to kill time during moments of leisure (S9).

The most disagreed items, on the contrary, reflect the level of divergence among the five personas on these statements, and thus constitute the most discriminating perspectives differentiating the five factors. The Z-scores range from 1.047 (S34) to 0.586 (S12). Among areas of disagreement (as rank-ordered) are practices of being attentive to latest updates in apps (S34), how they respond to having left the phone behind them (S24), keeping the device within easy reach or view (S23), and following the latest smartphone releases (S37). They disagree on whether smartphone use reduces their routine communication with close friends (S21), and differ on their habits of casual browsing the phone (S48). They deviate from one another on their perception of the smartphone as a digital companion (S44) and their practice of making switch to the latest phone releases (S39). There is a noticeable gap in their technical know-how with the smartphone (S38) as well as their inquisitiveness about mobile apps (S35). They also diverge (from 5 to 0) on whether they feel that too much time is spent on the smartphone (S12).

### Factor (persona) 1: the mainstreamer/typical user

Factor 1, which comprises 21 (five male vs. 16 female) participants, has an eigenvalue of 25.21 (composite reliability = 0.988) and explains 38% of the variance, far more than any of the other factors. They are leading all other groups in the number of self-reported hours on the smartphone. They place the most value on the smartphone as their private territory (29: + 5), and are in the habit of phone-checking prior to bed (20: + 5) as well as flipping through the phone once every few minutes (14: + 4). Time goes by quickly when they are connected (45: + 3), and they often regret over the amount of time the phone consumes them (12: + 2), resulting in a love-hate relationship with the device (11: + 2). They primarily consider value for the money in purchasing new phones (33: + 2), and do not attend to the latest technological developments or app releases (34: − 4; 37: − 4). They are not brand loyalists (36: − 5), and care the least about switching to the latest model when new phones are available (39: − 5).

If the smartphone is left behind, they feel an urge to go back and retrieve it before resuming normal activities (24: + 2). Among all personas, they have the least of self-control over use and time expended on the smartphone (13: − 4). Together with Persona 5, sentiments in Persona 1 are most likely to be disposed to technicalities surrounding the smartphone: they are inattentive to new phone releases (37: − 4) or technical features of apps (34: − 4); they admit no expertise about smartphone technologies or apps (38: − 3); and they rarely engage in searching new apps or their features (35: − 3). Users in this group are the least brand-aware (36: − 5), and they are in no rush to upgrade or switch to the latest phones (39: − 5). Noticeably, habitual pattern of use statistics provided by users suggests that Persona 1 surpasses all other personas in the number hours they are on the smartphone both on weekdays and weekend days.

### Factor (persona) 2: the disciplined conventionalists

Thirteen (four male and nine male) students make up this taxonomic category, which has an eigenvalue of 4.35 (composite reliability = 0.980) and explains 6% of the variance. While this cohort of students agree with their peers about the utility aspects of the smartphone, they are diametrically disposed to the other personas in many of the other dimensions, as illustrated by its between-factor correlation coefficients with the four other factors: − 0.63 (with factor 1); − 0.51 (with factor 3); − 0.47 (with factor 4); and − 0.51 (with factor 5). Of note is that the only negative correlation coefficients exist between factor 2 and the other factors. Members of this factor are the most reflexive about a number of ramifications that smartphone dependency may incur. This persona stands out above all others in often regretting what they think is the excessive amount of time they spend on the phone (12: + 5), even though their self-reported number of hours on weekdays and weekend does not indicate deviant usage patterns. They stand above all other groups in worrying that phone use has reduced the amount of communication they maintain with their close friends (21: + 3). This group shares with Persona 5 in feeling embarrassed at get-togethers with other people when everybody is busy checking phones without interpersonal interaction (17: + 3), and feeling offended by others looking at their phone while being talked to (15: + 3). They also take the lead, together with Persona 4, in trying to reduce their time on the smartphone (42: + 2). They differ from all other personas in slightly agreeing with the statement that more time on the smartphone may have distanced them further from friends and the external world (49: + 1).

They are the least receptive to attributing anthropomorphism to the smartphone among all five personas: they totally dispel the idea that the phone is like a right-hand pal to them (6: − 5) or it is a servant (3: − 5). They also stay away from any metaphoric reference to the smartphone as a personal agent (8: − 4), or a pet-like entity (7: − 4) or part of the family connections (7: − 3). They deny recognition to the phone the status of a close confidant like a twin brother or sister (31: − 3); nor are they willing to grant the device the role of a sibling (4: − 1) or a pal (44: − 1). Their responses to this set of items are the most consistent in distancing themselves from any anthropomorphic concession to the phone. This persona also exhibits the highest level of ambivalence among all personas about the seductive potential of the smartphone in admitting to a love-hate attitude (11: + 3).

### Factor (persona) 3: the casual fun-seekers

Factor 3 comprises six participants (evenly split along gender lines), with an eigenvalue of 3.77 (composite reliability = 0.960) and 6% explained variance. We call them casual fun-seekers because patterns of their smartphone engagement are dominated by entertainment-focused and casual information-seeking, as revealed in the three statements they ranked the highest (2: + 5; 48: + 5; 9: + 4). Together with students in Persona 4, they are hypo-connectors because the average amount of daily time they spend on the smartphone falls on the lower end. Similar to their peers in Factor 1, they often don’t feel how quickly their time on the phone goes by (45: + 3). They are more inclined than other groups in perceiving the phone merely as a tool—one that they can function without in accomplishing daily tasks (28: + 3). On the other hand, they are the most unlikely of all groups to think that phone use has affected their ability to communicate with others alternatively (18: − 4), or having the phone by their side can distract them from concentrating in class (15: − 4).

Contrary to Persona 2, students in this persona are more likely to assign anthropomorphic traits to the smartphone, such as a reliable assistant (50: + 4), a digital caretaker and aide (1: + 4), a good pal (44: + 2), a sibling (4: + 1), a personal assistant (8: + 1), or even a member of the extended family (7: + 1). They are also the most resistant to brand loyalty (36: − 5) and personalizing the phone (32: − 3). They are the least volatile to nomophobia, because they fare the best in keeping the phone out of sight (23: − 5) and don’t feel upset or uneasy if they leave it behind (24: − 3). With regard to emotional outcomes, this persona is rather consistent in denying that smartphone use has negatively impacted their ability to communicate with friends (21: − 2) or created distance between them and the outside world (49: − 4).

### Factor (persona) 4: the inquisitive nerds

This factor, with an eigenvalue of 2.91 (composite reliability = 0.923), comprises the smallest number (n = 3) of members, and accounts for 4% of the variance. Noticeably, the male-only persona is the most distanced from other factors, showing the lowest correlation coefficients (ranging from 0.30 to 0.39) with all other four personas. Similar to Persona 3, this is a hypo-connected group in the amount of time they spend on the smartphone. This persona places the most value to the multifunctional, utilitarian aspects of the smartphone (10: + 5), and considers the phone their private domain that should not be intruded upon (29: + 5). They stand out from all other groups in their innovativeness and technology-oriented approach to the smartphone. They try to stay abreast with latest apps and want to be the first to try out new features (34: + 4); they also make an effort in developing the know-how and technical skills in tapping into new apps and smartphone technologies (30: + 3). Students adhering to this persona perceive themselves being ahead of their peers in understanding smartphone-related technologies (38: + 2); and they are attentive to latest phone releases (37: + 2). While being sensitive to pricing in purchasing new phones (33: + 4), they are leading all other groups in their urge to upgrade to the newest phone (39: + 1) but not necessarily in chasing a luxurious brand name (36: − 3). The fact that this is not ranked higher is probably due to the financial constraints of being a student. Unsurprisingly, these students are also leading their peers in the willingness to personalize their phones (32: + 2) and constantly searching for and installing new apps (35: + 2).

Unlike those in other groups, these students display a mixed approach to nomophobia: while it is relatively important for them to keep their phone within sight (23: + 1), they are not in a desperate mood in retrieving their phone if it is left behind (24: − 2). They align themselves with those in Persona 3 in not showing concerns about ontological consequences of smartphone use in terms of affecting their ability to communicate with others (18: − 3; 21: − 4) or creating gap between them and the rest of the world (49: − 4). But they differ from Persona 3 in that they steer away from impregnating anthropomorphic attributes to the smartphone (7: − 1; 8: − 1; 5: − 2; 4: − 2; 31: − 2; 3: − 3; 33: − 5). This cohort also attaches the most importance to the phone being a platform for presenting themselves (46: + 4).

### Factor (persona) 5: the sentient pragmatists

Our final category includes eight users, and its factorial structure bears an eigenvalue of 2.48 (composite reliability = 0.970) and 4% explained variance. Its gender makeup (7 female vs. 1 male) almost falls on the opposite end of Persona 4. It is worth noting that the composite components of this factor overlap sizably with factors 1 and 3, but differ the most from factor 4. This is reflected in its inter-factor correlation coefficient scores being the highest with Factors 1 to 3, but the lowest with Factor 4.

In bearing semblance to Persona 1 (but at a higher degree), these students display the strongest urge to go back and retrieve the phone if it is left back (24: + 3), and they are the most disposed to feel uneasy when the phone is out of sight (23: + 2). While losing the phone would not be such a great deal for other groups except Persona 1 (+ 1), this would be a more of a blow to this group (26: + 2). In a similar but more prominent pattern to Persona 3, anthropomorphic traits of the smartphone are ranked the highest relative to other groups with regard to comparing the phone to a personal assistant (50: + 4), a digital caretaker (1: + 4), a personal assistant (8: + 2), and a member of the extended family (7: + 2).

Persona 5 concurs with Persona 2 on the courtesy and etiquette of conventional face-to-face communication (albeit at a more elevated level): they get offended if others eye at the smartphones while listening to them (16: + 4), and they feel embarrassed if people sit together checking their phones instead of talking to one another (17: + 3). But they disagree with Persona 2 in that phone use has negatively impacted their ability to communicate with friends (21: − 2 vs. + 3). Additionally, they are positioned at the opposite end vis-à-vis Persona 4 in their technological orientation and tech savviness.

### Emerging themes with open-ended answers

In explaining their sorting choices, participants across all five personas consistently cited everyday experiences of engagement with, and individual perceptions of the smartphone, as driving their decision-making, which aligns squarely with what this research strives for. While stressing the utility nature of the device in enabling multiple tasks and functionalities, the students in Persona 1 uniformly deny any intimate relationship with the phone and distance themselves from personifying it. Many explicitly acknowledge that the smartphone has changed their habits in everyday life, such as “aimless browsing” during moments of leisure and regularly checking the phone for fear of missing information. Also conspicuously highlighted is the importance the smartphone being their private territory, as revealed in one student’s comment “I am extremely repugnant to those who peep into my personal stuff”. Their ambivalence toward the phone is prominently noted, as illustrated by one student who said “I sometimes feel annoyed by the smartphone, because I feel an urge to play around with it for no purpose in particular” and another with the remark “while logging onto my phone, it drags on and on so much that this often results in procrastination with my other errands.” Perceived lack of control in front of what some called the “temptation” of the smartphone was mentioned repeatedly, but many made a point in underscoring that the phone facilitates, rather than hampers communication with their close ones, especially family members. A few students discussed their perceived sense of security in the presence of the smartphone, to which one student remarked: “even the touch of the phone puts me at ease when I wake up at night”.

Among the twelve participants who volunteered detailed comments in Persona 2, there is an unmistakable prevailing emphasis on the double-edged nature of the smartphone: it is useful but can be addictive; it facilitates doing things but is not indispensable; it has lots to offer but should not dictate what they do. This thematic line is remarkably consistent interconnecting all comments—a pattern that differentiates this persona from the others. Other discursive threads include conscientious efforts in controlling the amount of time they expend on the phone, intentional balance between smartphone-based entertainment and study, the need to ponder the potential negative impact of the smartphone on spiritual and physical well-being, and the importance of maintaining conventional modes of communication.

As for Persona 3, a recurring theme among the participants is the entertaining value of smartphone-enabled apps, which “spice up my life” in one student’s words, and “provide the fodder for the daily chitchat” for another student. Participants pointed out that the phone brings about a lot of convenience to their life, but they could function in the absence of the phone, albeit with “nuisances.” Two students highlighted the “smartness” of the phone in that it would be a waste if they “did not exploit the advanced technical features.” But they denied that the phone has distanced them from their close friends and family members. Worth noting is that Persona 3 deviates from all other personas in having none of the participants disavowing anthropomorphism in the remarks (although they did not specifically embrace that either). In contrast, two (out of three) students in Persona 4 made a point in registering their “loathness” to the personification of (alongside excessive dependence on) the smartphone. A consistent configuration of discursive focus among all three students in Persona 4 is their infatuation with technical innovativeness and specific features. Finally, as regards Persona 5, three students mentioned their “painful” memories of previous losses of the phone, which probably contributes to the higher level of nomophobia as measured in their Q sorts. Moreover, participants’ exposition avers that the smartphone has not adversely impacted their interpersonal communication with friends and family, and a few expressed “disgust” at people who are distracted by the phone in face-to-face conversations.

## Conclusions

College students and young adults are spearheading the frontlines of an emerging global mobile culture through appropriating smartphone technologies and negotiating lived experiences [1, 71]. Inquiring into the dynamics of college students’ relationships with their smartphones therefore has much to contribute to our understanding of the rapidly evolving youth-driven mobile lifestyles. This research set out to investigate students’ multidimensional relationships with the smartphone through the theoretical perspectives of mobile attachment in combination with the analytical power of Q methodology. The concourse of *subjective communicability* (Q statements) [56], which was derived through an extensive review of current research literature coupled with pilot interviews and informal surveys, covers a wide-ranging scope of topics and issues in broad relevance to user attachment to the smartphone. In comparison with previous research endeavors which tend to focus on a limited number of conceptual areas, our 50-item tool provides the advantage of an all-encompassing, holistic approach to many related issues within one framework. Our findings thus not only shed light on the rapidly evolving, contested formations of the youth-driven mobile lifestyles life in China but also expand our understanding of the nuanced nature of user-device attachment with the smartphone.

First of all, the procedural design of the forced-choice Q sort structure affords us the added vantage point of holistically (that is, a gestalt view) comparing rank orders of the diverse aspects of mobile attachment which have been most typically examined in variable-analytical approaches in previous research settings. A core set of consensus statements identified by Q methodology’s by-person factor analysis confirms the central and pervasive role of the smartphone in the daily lived experiences of college students from information-seeking, entertainment, to an enhanced sense of security. Moreover, it comes as no surprise that the smartphone has effectively established itself as mega-platform beyond the role as a mere tool of communication. As a result, various relationships of attachment have developed between the device and its users. In this regard, the smartphone, by incorporating multiple functionalities into a unified platform, has in essence consolidated an integrated environment of polymedia [72] or metamedia [73].

Results in our study demonstrate that there is a wide range of variation in psycho-emotional perception of the smartphone. The Q statements that caused the most divergence include attentiveness to new apps and updates as well as new phone releases, psychological responses to smartphones being out of reach or out of sight (nomophobia), the extent to which one engages in aimless or pleasure scrolling on the phone, and interest in the nerdiness of smartphone-related technicalities. More specifically, the typology of five personas demonstrates the diversified nature of user-device dynamics with regard to smartphone use. Mainstream users, disciplined conventionalists, casual fun-seekers, inquisitive nerds, and sentient pragmatists exhibit different approaches to engaging with the smartphone.

Mainstream users (Persona 1) tend to spend more time on their smartphones and are sensitive to privacy issues, and they are pragmatists in caring more about the functionalities and value-for-the-money than brand; but they are aligned the most closely with commonly recognized symptoms of smartphone addiction as measured in the multiplicity of Q sorts from excessive use to dependency to control [74]. This is also corroborated by their recognized love-hate relationship with the device. Diametrically antithetical to the other groups on most dimensions, disciplined conventionalists (Persona 2) make the most conscientious effort in controlling the amount of time they stay on the smartphone and are the most reflexive on the addictive nature of the gadget, even though their actual time on the smartphone stays average. They are the most repugnant to phubbing and the least susceptible to anthropomorphic attributions as regards smartphone use.

Casual fun-seekers (Persona 3), on the other hand, primarily use the smartphone for entertainment and leisure, and they are the most averse to nomophobia. They are also the most susceptible to anthropomorphism and emotional connotations of the smartphone, while they are also the most adamant among all peers in maintaining that smartphone use has done little in detaching them from real-world connections and communications. Inquisitive nerds (persona 4), which are male-dominated, display a palpable love for the smartphone as a technological wonder. They lead all other groups in their tendency to personalize the smartphone, clearly for its technological forefronts rather than its anthropomorphic appeal. Sentient pragmatists (Persona 5) are quite opposite to inquisitive nerds in that they are female-dominated, and are the least tech-savvy. Meanwhile, they lead all groups in symptoms of nomophobia, and proclivity to anthropomorphic attribution. Their smartphone use is entertainment-centric, and they take the most offense at phubbing.

It must be noted that, despite the distinctiveness of the Q factor structure, there exist convoluted patterns of overlapping among the different personas. The clusters of conceptual concentrations we have identified along the dimensions of utility, functionality, habituation, anthropomorphism, nomophobia, techno-innovativeness, and their interrelated configurations into the different personas are worthy of further scrutiny in future research, especially as they relate to the respective psycho-emotional attachment and other outcomes.

Findings are best evaluated in the context of their limitations. As an intensive form of analysis that uses small numbers of participants, Q methodology offers insight over representations of discourses as they exist within a larger population [58]. We make no claim that the subjects of our investigation are representative of the overall college student population, or even college students in China. Nonetheless, as a robust approach, Q analysis thrives in highly reliable factors scores and detecting patterns of relationships among arrays of statements using a small number of participants. The utility of Q methodology as demonstrated in our research can be productively applied in offering alternative and competing insight over user-device relationships targeting diverse demographics and user groups. There are also notable demarcations in use patterns and user perceptions along gender lines that should be important areas of further research.

“Finite diversity” in Q methodology means that only a range of viewpoints can be covered in the Q statements [63]. Thus there is no guarantee that all factors have been located in any analysis. Our study is inherently limited by the number of statements we can include. Although it is useful to identify the extensive list of consensus statements in our findings, we suggest that future research adopt a more divergent concourse of statement items in order to bolster the discriminant power of the Q sample in dissecting individual variations. Moreover, we do not claim that the typology revealed in our study is universally applicable to the full spectrum of smartphone use, or even to college students in general. Nor are we the view that the typology will remain stable over time, even among the same participants. Therefore, to what extent the patterns of our findings converge or diverge across different user bases in cross-cultural contexts call for interrogation from future research.

## Data Availability

The data that support the findings of this study are available from the corresponding author, CD, upon reasonable request.

## References

[1] Vanden Abeele MM (2016). Mobile youth culture: a conceptual development. Mob Media Commun.

[2] Goggin G (2021). Apps: from mobile phones to digital lives.

[3] Kang S, Jung J (2014). Mobile communication for human needs: a comparison of smartphone use between the US and Korea. Comput Human Behav.

[4] Rosaline S, Johnson S (2020). Continued usage and dependency of smartphones. Int J Cyber Behav Psychol Learn.

[5] Chan-Olmsted S, Xiao M (2019). Factors affecting smartphone dependency of media consumers. Int J Mobile Commun.

[6] Bowlby J (1969). Attachment and loss.

[7] Feeney BC, Collins NL, Rholes WS, Simpson JA (2004). Interpersonal safe haven and secure base caregiving processes in adulthood. Adult attachment: theory, research, and clinical implications.

[8] Ainsworth MDS, Blehar MC, Waters E, Wall SN (2015). Patterns of attachment: a psychological study of the strange situation.

[9] Bröning S, Wartberg L (2022). Attached to your smartphone? A dyadic perspective on perceived partner phubbing and attachment in long-term couple relationships. Comput Hum Behav.

[10] Musetti A, Manari T, Billieux J, Starcevic V, Schimmenti A (2022). Problematic social networking sites use and attachment: a systematic review. Comput Hum Behav.

[11] Diefenbach S, Borrmann K. The Smartphone as a Pacifier and its consequences: young adults' smartphone usage in moments of solitude and correlations to self-reflection. In: Proceedings of the 2019 CHI conference on human factors in computing systems; 2019. p. 1–14.

[12] Melumad S, Pham MT (2020). The smartphone as a pacifying technology. J Consum Res.

[13] Parent N, Bond TA, Shapka JD (2021). Smartphones as attachment targets: an attachment theory framework for understanding problematic smartphone use. Curr Psychol.

[14] Belk R (2016). Extended self and the digital world. Curr Opin Psychol.

[15] Belk RW (2013). Extended self in a digital world. J Consum Res.

[16] Sheth JN, Solomon MR (2014). Extending the extended self in a digital world. J Mark Theory Pract.

[17] Harkin LJ, Kuss D (2021). “My smartphone is an extension of myself”: a holistic qualitative exploration of the impact of using a smartphone. Psychol Pop Media.

[18] Wehmeyer K (2008). User-device attachment? Scale development and initial test. Int J Mobile Commun.

[19] Park CS (2019). Examination of smartphone dependence: functionally and existentially dependent behavior on the smartphone. Comput Hum Behav.

[20] Park CS, Kaye BK (2019). Smartphone and self-extension: functionally, anthropomorphically, and ontologically extending self via the smartphone. Mob Media Commun.

[21] Ross MQ, Bayer JB (2021). Explicating self-phones: dimensions and correlates of smartphone self-extension. Mob Media Commun.

[22] Carolus A, Binder JF, Muench R, Schmidt C, Schneider F, Buglass SL (2019). Smartphones as digital companions: characterizing the relationship between users and their phones. New Media Soc.

[23] Rodríguez-Torrico P, Prodanova J, San-Martín S, Jimenez N (2020). The ideal companion: the role of mobile phone attachment in travel purchase intention. Curr Issues Tour.

[24] Fullwood C, Quinn S, Kaye LK, Redding C (2017). My virtual friend: a qualitative analysis of the attitudes and experiences of Smartphone users: implications for Smartphone attachment. Comput Hum Behav.

[25] Marchant C, O’Donohoe S (2019). Homo prostheticus? Intercorporeality and the emerging adult-smartphone assemblage. Inf Technol People.

[26] Wang W (2017). Smartphones as Social Actors? Social dispositional factors in assessing anthropomorphism. Comput Hum Behav.

[27] Jarvenpaa SL, Lang KR (2005). Managing the paradoxes of mobile technology. Inf Syst Manag.

[28] Ter Hoeven CL, van Zoonen W, Fonner KL (2016). The practical paradox of technology: the influence of communication technology use on employee burnout and engagement. Commun Monogr.

[29] Trub L, Barbot B (2016). The paradox of phone attachment: Development and validation of the Young Adult Attachment to Phone Scale (YAPS). Comput Hum Behav.

[30] McDaniel B (2016). "Technoference”: everyday intrusions and interruptions of technology in couple and family relationships. Psychol Pop Media Cult.

[31] McDaniel BT, Drouin M (2019). Daily technology interruptions and emotional and relational well-being. Comput Hum Behav.

[32] Bhattacharya S, Bashar MA, Srivastava A, Singh A (2019). Nomophobia: no mobile phone phobia. J Fam Med Prim Care.

[33] Nie J, Wang P, Lei L (2020). Why can't we be separated from our smartphones? The vital roles of smartphone activity in smartphone separation anxiety. Comput Human Behav.

[34] Rodríguez-García A-M, Moreno-Guerrero A-J, Lopez BJ (2020). Nomophobia: an individual’s growing fear of being without a smartphone—a systematic literature review. Int J Environ Res Public Health.

[35] Yildirim C, Correia A-P (2015). Exploring the dimensions of nomophobia: development and validation of a self-reported questionnaire. Comput Hum Behav.

[36] Al-Saggaf Y, O'Donnell SB (2019). Phubbing: perceptions, reasons behind, predictors, and impacts. Hum Behav Emerg Technol.

[37] David ME, Roberts JA (2017). Phubbed and alone: phone snubbing, social exclusion, and attachment to social media. J Assoc Consum Res.

[38] Erzen E, Odaci H, Yeniçeri İ (2019). Phubbing: which personality traits are prone to phubbing?. Soc Sci Comput Rev.

[39] Eyal N (2014). Hooked: how to build habit-forming products.

[40] Oulasvirta A, Rattenbury T, Ma L, Raita E (2012). Habits make smartphone use more pervasive. Pers Ubiquit Comput.

[41] Bayer JB, LaRose R, Verplanken B (2018). Technology habits: progress, problems, and prospects. The psychology of habit: theory, mechanisms, change, and contexts.

[42] Wood W, Rünger D (2016). Psychology of habit. Annu Rev Psychol.

[43] Anshari M, Alas Y, Sulaiman E (2019). Smartphone addictions and nomophobia among youth. Vulnerable Child Youth Stud.

[44] Carvalho LF, Sette CP, Ferrari BL (2018). Problematic smartphone use relationship with pathological personality traits: systematic review and meta-analysis. Cyberpsychology (Brno)..

[45] De-Sola Gutiérrez J, Rodríguez de Fonseca F, Rubio G (2016). Cell-phone addiction: a review. Front Psychiatry.

[46] Derevensky JL, Hayman V, Gilbeau L (2019). Behavioral addictions: excessive gambling, gaming, Internet, and smartphone use among children and adolescents. Pediatr Clin.

[47] Duke É, Montag C (2017). Smartphone addiction, daily interruptions and self-reported productivity. Addict Behav Rep.

[48] Enez Darcin A, Kose S, Noyan CO, Nurmedov S, Yılmaz O, Dilbaz N (2016). Smartphone addiction and its relationship with social anxiety and loneliness. Behav Inf Technol.

[49] Wolniewicz CA, Tiamiyu MF, Weeks JW, Elhai JD (2018). Problematic smartphone use and relations with negative affect, fear of missing out, and fear of negative and positive evaluation. Psychiatry Res.

[50] Yu S, Sussman S (2020). Does smartphone addiction fall on a continuum of addictive behaviors?. Int J Environ Res Public Health.

[51] Pancani L, Preti E, Riva P (2020). The psychology of smartphone: the development of the smartphone impact scale (SIS). Assessment.

[52] Altamimi TN, Alex J, Khan MM, Nair BV (2020). Development and validation of a Smartphone Impact Scale among healthcare professionals. J Taibah Univ Med Sci.

[53] Enez Ö, Yalçınkaya-Alkar Ö (2021). Assessing mobile phone attachment: validation of the mobile attachment questionnaire in Turkish University students and examination of related variables. Psychol Rep.

[54] Stephenson W (1953). The study of behavior: Q-technique and its methodology.

[55] Previte J, Pini B, Haslam-McKenzie F (2007). Q methodology and rural research. Sociol Rural.

[56] McKeown B, Thomas DB (2013). Q methodology.

[57] Ramlo S (2016). Mixed method lessons learned from 80 years of Q methodology. J Mixed Methods Res.

[58] Brown SR (1980). Political subjectivity: applications of Q methodology in political science.

[59] Simons J (2013). An introduction to Q methodology. Nurse Res.

[60] Stenner P, Stainton RR, Todd Z, Nerlich B, McKeown S, Clarke D (2004). Q methodology and qualiquantology: the example of discriminating between emotions. Mixing methods in psychology: the integration of qualitative and quantitative methods in theory and practice.

[61] Goldman I (1999). Q methodology as process and context in interpretivism, communication, and psychoanalytic psychotherapy research. Psychol Rec.

[62] Watts S, Stenner P (2005). Doing Q methodology: theory, method and interpretation. Qual Res Psychol.

[63] Stainton Rogers R, Smith JA, Harré R, Van Langenhove L (1995). Q methodology. Rethinking methods in psychology.

[64] Addams H, Proops JL (2000). Social discourse and environmental policy: an application of Q methodology.

[65] Fortier A, Burkell J (2018). Display and control in online social spaces: towards a typology of users. New Media Soc.

[66] Zeller F, O’Kane J, Godo E, Goodrum A (2014). A subjective user-typology of online news consumption. Digit J.

[67] Newman I, Ramlo S, Tashakkori A, Teddlie C (2010). Using Q methodology and Q factor analysis in mixed methods research. Sage handbook of mixed methods in social and behavioral research.

[68] Clarke V, Braun V, Hayfield N, Smith JA (2015). Thematic analysis. Qualitative psychology: a practical guide to research methods.

[69] Karnowski V, Jandura O (2014). When lifestyle becomes behavior: a closer look at the situational context of mobile communication. Telematics Inform.

[70] Ling R (2004). The mobile connection: the cell phone's impact on society.

[71] Dai C, Tai Z, Ni S (2021). Smartphone use and psychological well-being among college students in China: a qualitative assessment. Front Psychol.

[72] Madianou M (2014). Smartphones as polymedia. J Comput-Mediat Commun.

[73] Humphreys L, Karnowski V, von Pape T (2018). Smartphones as metamedia: a framework for identifying the niches structuring smartphone use. Int J Commun.

[74] Luk TT, Wang MP, Shen C, Wan A, Chau PH, Oliffe J, Viswanath K, Chan SS-C, Lam TH (2018). Short version of the Smartphone Addiction Scale in Chinese adults: psychometric properties, sociodemographic, and health behavioral correlates. J Behav Addict.

